# Glutamate Concentration in the Medial Prefrontal Cortex Predicts Resting-State Cortical-Subcortical Functional Connectivity in Humans

**DOI:** 10.1371/journal.pone.0060312

**Published:** 2013-04-03

**Authors:** Niall W. Duncan, Christine Wiebking, Brice Tiret, Malgoranza Marjańska, Dave J. Hayes, Oliver Lyttleton, Julien Doyon, Georg Northoff

**Affiliations:** 1 Mind, Brain Imaging and Neuroethics Research Unit, University of Ottawa Institute of Mental Health Research, Ottawa, Canada; 2 Department of Biology, University of Carleton, Ottawa, Canada; 3 Department of Biology, Freie Universität, Berlin, Germany; 4 Functional Neuroimaging Unit and Department of Psychology, University of Montreal, Montreal, Canada; 5 Center for Magnetic Resonance Research and Department of Radiology, University of Minnesota, Minneapolis, Minnesota, United States of America; Hangzhou Normal University, China

## Abstract

Communication between cortical and subcortical regions is integral to a wide range of psychological processes and has been implicated in a number of psychiatric conditions. Studies in animals have provided insight into the biochemical and connectivity processes underlying such communication. However, to date no experiments that link these factors in humans *in vivo* have been carried out. To investigate the role of glutamate in individual differences in communication between the cortex – specifically the medial prefrontal cortex (mPFC) – and subcortical regions in humans, a combination of resting-state fMRI, DTI and MRS was performed. The subcortical target regions were the nucleus accumbens (NAc), dorsomedial thalamus (DMT), and periaqueductal grey (PAG). It was found that functional connectivity between the mPFC and each of the NAc and DMT was positively correlated with mPFC glutamate concentrations, whilst functional connectivity between the mPFC and PAG was negatively correlated with glutamate concentration. The correlations involving mPFC glutamate and FC between the mPFC and each of the DMT and PAG were mirrored by correlations with structural connectivity, providing evidence that the glutamatergic relationship may, in part, be due to direct connectivity. These results are in agreement with existing results from animal studies and may have relevance for MDD and schizophrenia.

## Introduction

Effective communication between cortical and subcortical regions is likely essential for most psychological functions, as well as for the intrinsic brain networks seen at rest. For example, recent reports have underscored the importance of such communication in higher order emotional functioning as well as in more fundamental reward and aversion-related processing [1]–[3]. Detailed knowledge of the systems involved in such cortical-subcortical communication has been obtained through many years of animal studies which have provided an increasing understanding of the networks and numerous neurotransmitters involved, highlighting, for example, the roles of the medial prefrontal cortex (mPFC) and transmitters such as glutamate [4]–[7].

Given the obvious experimental challenges, *in vivo* studies of cortical-subcortical connections in humans have, in contrast, been less common. However, advances in imaging technology now make it possible for studies to be carried out that link structure, function, and biochemistry [8]–[13]. In addition to advancing the basic understanding of the brain, such research may be of particular interest as changes in cortical-subcortical communication have been proposed to underlie a variety of psychiatric disorders, including depression and schizophrenia [6], [14], [15]. In the latter context, glutamatergic communication presents a promising target as glutamate has been implicated in each of these disorders [16]–[18].

The current study aimed to undertake a preliminary investigation of the biochemical underpinnings of inter-individual differences [19] in resting-state cortical-subcortical communication in humans, focusing on glutamate in the mPFC. The mPFC was chosen as the seed region based, firstly, upon extensive evidence from animal studies that this region is connected to a large set of subcortical regions [20], [21] via glutamatergic processes [22]–[26]; secondly, upon prior results in humans showing that glutamate levels in the mPFC can be related to task-based connectivity between this region and others, including subcortical ones [10]; and thirdly, upon the apparently key role that this region plays in psychiatric conditions linked to the glutamatergic system [6], [14], [15], [27]. Using a combination of resting-state fMRI, diffusion-tensor imaging (DTI), and magnetic resonance spectroscopy (MRS), functional and structural connectivity between the mPFC (centred on the perigenual anterior cingulate; pgACC) and three selected subcortical regions were measured. These regions were the nucleus accumbens (NAc), dorsomedial thalamus (DMT), and periaqueductal grey (PAG). Given the exploratory nature of the investigation, this small number of target regions was used to minimise the problem of multiple comparisons. The target regions chosen were selected, firstly, as anatomical connections between each of them and the mPFC have been shown across a range of species [28]–[32], and secondly due to these connections being known, through work in both humans and non-human animals, to involve glutamate to some degree [22]–[26], [33]. In addition, the regions were selected as they have been implicated in disorders such as schizophrenia and depression [6], [34]–[36] and as they are of interest as components of what has been termed the core and para-core limbic systems that underlie fundamental stimulus processing [14], [37], [38].

MRS was used to measure resting-state glutamate and glutamine concentrations within the mPFC. Glutamatergic measures were then correlated with the degree of resting-state functional connectivity (FC) between the mPFC and each of the target regions. DTI was used to establish if the FC observed was related to structural connectivity, and thus direct inter-regional communication. In order to provide some degree of specificity to any observed relationship between mPFC glutamatergic measures and FC, glutamatergic MRS measures from the left insula were used as regional control values.

Although the study was mainly exploratory, it was hypothesised that glutamate levels in the mPFC would be positively related to resting-state functional connectivity. This was based upon the prior knowledge that glutamate-related connections between the mPFC and the subcortical regions of interest exist [22]–[26], [33] and that glutamate has been implicated in inter-regional communication *in vivo* in humans [10]. As the relationship between functional and structural connectivity is complex [8], [39], no hypotheses were proposed in this regard.

## Methods

### Subjects

Twenty-eight healthy subjects were scanned using fMRI and MRS at two different MRI centres (fMRI – Montreal Neurological Institute, McGill University; MRS – Unité de neuroimagerie fonctionelle, Université de Montréal). Siemens 3T Trio MRI scanners were used at both locations. The data from six subjects were rejected due to excessive head movement during the fMRI scan, four subjects due to deviance from the task, and from five subjects due to poor quality MRS data. The analyses were carried out on the rest of the data from 13 subjects (5 females; mean age = 22.1 years, range: 18–32 years). The mean time between scans for the subjects to be included was 3.6 days (range: 1–10 days). Subjects were screened for psychiatric or neurological disorders, recreational drug use, and depression, the latter using the Beck Depression Inventory-II with a cut-off score of four [40].

### Ethics Statement

All subjects gave their written informed consent and were compensated financially for their participation. Approval for the study was obtained from the ethics committees at both McGill University and the Université de Montréal.

### MRS

Single voxel edited 1H MR spectra were acquired using the MEGA-PRESS method [41], [42] with a body coil transmit and 12-channel receive headcoil. Using a high resolution T1-weighted anatomical image (MPRAGE; FOV = 205×205 mm2; spatial resolution = 1×1×1 mm3; TE = 3.02 ms; TR = 2000 ms; flip angle = 5°), volumes of interest (VOI) were located in the mPFC and the left insula (see Figure S1a for locations). In order to achieve consistent VOI positioning, placement was done by the same investigator for all subjects according to easily identifiable anatomical landmarks: mPFC VOIs (48×21×21 mm3) were placed anterior to the genu of the corpus callosum, parallel to the AC-PC plane; left insula VOIs (23×48×27 mm3) were aligned with the line of the insula cortex in an anterior-posterior direction with the most anterior edge of the VOI aligned to the anterior limit of the insula.

First- and second-order shim terms were adjusted using FASTMAP with echo-planar imaging readout [43]. MRS data were acquired using a MEGA-PRESS sequence [41], [44] with double-banded pulses used to simultaneously suppress water signal and edit the γ–CH2 resonance of GABA at 3 ppm. Additional water suppression using variable power with optimized relaxation delays (VAPOR) and outer volume suppression (OVS) techniques [45] was optimized for the human 3 T system and incorporated prior to MEGA-PRESS. The final spectra were obtained by subtracting the signals from alternate scans with the selective double-banded pulse applied at 4.7 ppm and 7.5 ppm (‘EDIT OFF’) and the selective double-banded pulse applied at 1.9 ppm and 4.7 ppm (‘EDIT ON’). MEGA-PRESS data were acquired in four interleaved blocks of 32 (‘EDIT OFF’, ‘EDIT ON’) scans each with frequency adjustment between each block. FIDs were stored separately in memory for individual frequency and phase correction using the tCr signal at 3.03 ppm, as well as correction for residual eddy-current using unsuppressed water signal obtained from the same voxel.

Difference spectra were analyzed using LCModel 6.2-1A [46], [47] using the basis set which included an experimentally measured metabolite-nulled macromolecular spectrum (average from 10 subjects) and the experimentally measured spectra from 100 mM phantoms of N-acetylaspartate (NAA), gamma-amino butyric acid (GABA), glutamate (Glu), and glutamine (Gln) with pH adjusted to 7.2 and at 37°C. The LCModel fitting was performed over the spectral range from 0.5 to 4.0 ppm. No baseline correction, zero-filling, or apodization functions were applied to the in vivo data prior to LCModel analysis.

Only results with the Cramer-Rao lower bounds below 20% were included in the analysis. Concentrations with CRLB >20% were classified as not detected. Although the estimated correlation coefficients, derived from a standard least-squares variance-covariance matrix of LCModel analysis, indicated a strong negative correlation between Glu and Gln, both Glu and Gln were reliably quantified in all subjects. Combined Glu+Gln (Glx), plus individual Glu and Gln are reported (see Table S1 for metabolite concentrations and CRLB values). LCModel quantification of the representative spectrum is shown in Figure S1b.

The mPFC, centred on the perigenual anterior cingulate cortex (pgACC), was the target region for the study and the left insula was used as a regional specificity control. The first step in each analysis was to use the combined Glu+Gln (Glx) concentrations, as a ratio to NAA, for correlation with functional connectivity (Glx/NAA). In a second step, functional connectivity was correlated with the individual values for Glu/NAA and Gln/NAA.

### ROIs

**Figure 1:**
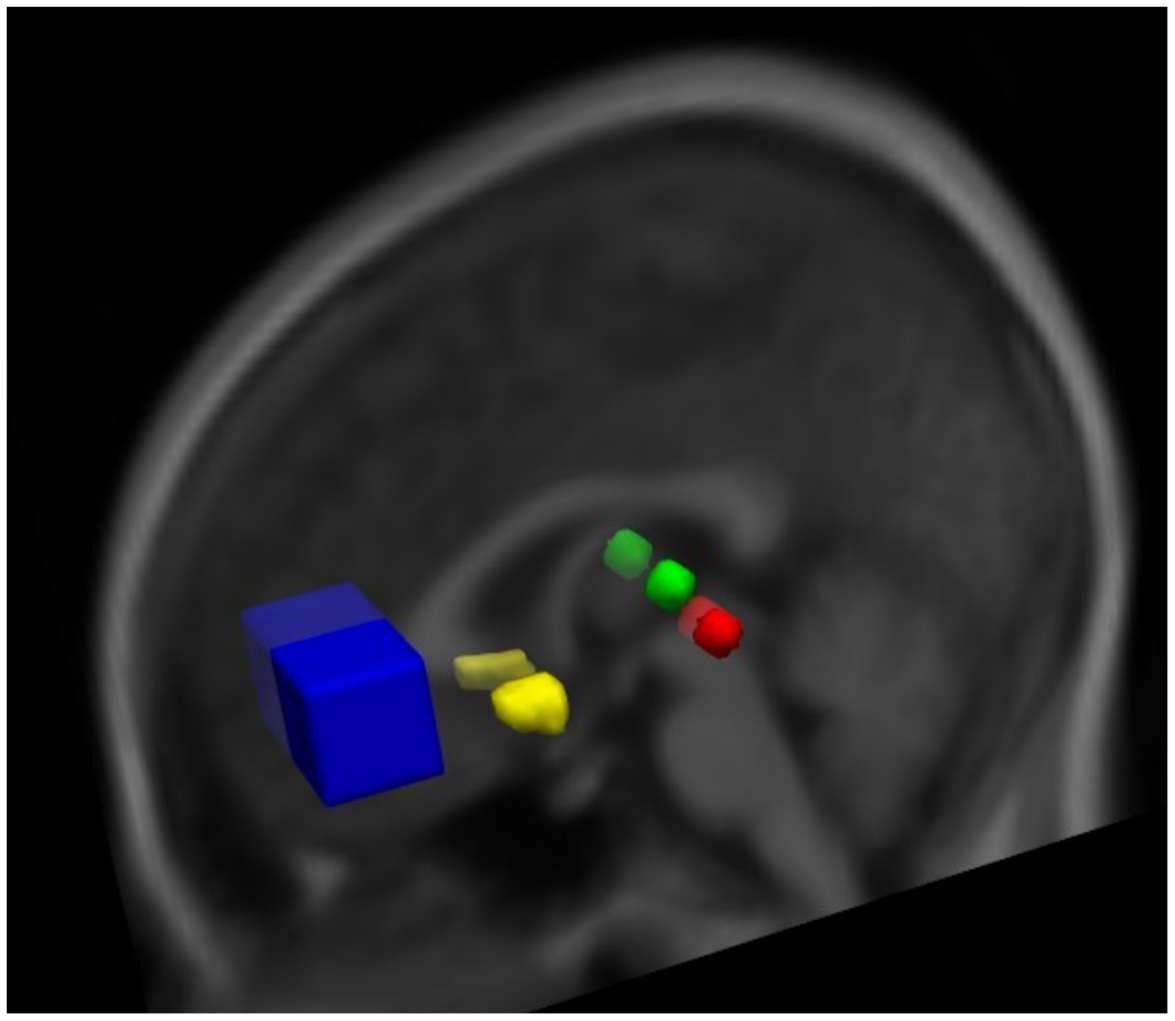
Region of interest locations. ROI placement displayed on group mean anatomical image in MNI standard space. mPFC - blue, NAc - yellow, DMT - green, PAG - red.

The mPFC MRS region was used as the seed ROI. Left and right NAc ROIs were defined as the NAc region from the Harvard-Oxford subcortical atlas (probability 0.5)(http://www.cma.mgh.harvard.edu/fsl_atlas.html). Spherical target ROIs with a radius of 4 mm were placed in the DMT and PAG bilaterally in standard space (MNI). Coordinates for the DMT ROIs (left = 12 −18 8; right = −10 −18 8) were obtained by identifying the thalamus on the mean group anatomical image using the Harvard-Oxford atlas and then visually centring the spheres within the dorso-medial portion. Coordinates for the PAG ROIs (left = −4 28 9; right = 4 −28 −6) were adapted from the literature [48]. See Figure 1 for ROI locations.

### fMRI

Functional EPI scans were acquired using a body coil transmit and 32-channel receive headcoil. Forty-seven slices aligned at −30° from the AC-PC plane and covering the whole brain were acquired per volume, with a total of 467 volumes being acquired (1060 s) over the task run (FoV = 205×205 mm^2^; spatial resolution = 3.2×3.2×3.2 mm^3^; TE = 25 ms; TR = 2270 ms; flip angle = 90°). The first five volumes were discarded. High-resolution T_1_-weighted anatomical image were also acquired (MPRAGE; FOV = 205×205 mm^2^; spatial resolution = 1×1×1 mm^3^; TE = 3.02 ms; TR = 2000 ms; flip angle = 5°).

The session consisted of two long eyes-open (EO) and two long eyes-closed (EC) periods (2×120 s, 212 volumes, alternating and counterbalanced across subjects; Figure S2a), that followed on from a sequence of short EO and EC blocks (255 volumes), the data for which were not analysed here. EC conditions were indicated by a single short tone and EO by a short double tone (100 ms and 2×100 ms, respectively). Small EO and EC icons were also displayed to inform subjects of the current condition in case of confusion. Subjects were observed using a simple camera setup to ensure that they followed the task instructions.

The processing of all fMRI data was carried out with the FSL suite of tools [49], [50]. Functional images were corrected for head movement, brain-extracted, high-pass filtered with a 100 s cut-off, and smoothed with a 6 mm at FWHM Gaussian kernel. Functional data was then variance normalised by dividing each voxel by its standard deviation over time. Non-brain tissue was removed from the anatomical images prior to segmentation into white matter (WM), grey matter (GM), and cerebrospinal fluid (CSF) maps using FAST [51]. Linear alignments between the functional images and the MNI template were calculated via the high-resolution anatomical image.

Masks corresponding to the mPFC and left insula MRS boxes were applied to the individual GM maps, thresholded at 0.95, to produce subject specific masks of GM within the MRS boxes (MRS-GM). The proportion of grey matter within the MRS box was included in all group-level analyses as a confounding variable. Following binarisation, these masks were transformed into individual functional space and used as ROIs for the functional connectivity analyses. Mean timecourses were extracted from within the MRS-GM ROIs (from the unsmoothed pre-processed data). These were then mean-centred, variance normalised, and split into the EO and EC conditions (Figure S2b).

Segmented WM and CSF maps were made into binary mask images using a threshold of 0.99 and then eroded by one voxel to ensure that there was no overlap between the masks and GM. These WM and CSF ROIs were transformed into functional space and the first eigenvariate of the timecourse in each ROI extracted for each subject (from the unsmoothed pre-processed data). These CSF and WM timecourses were included in the functional connectivity model as nuisance regressors along with the six head movement parameters obtained during pre-processing. These regressors were included to minimise the effect of head movement and physiological and other noise sources on the functional connectivity calculations.

A model consisting of the EO and EC timeseries from the mPFC (Figure S2b), along with the eight nuisance regressors, was constructed and entered into a multiple regression analysis with the variance normalised functional data [52]–[54]. In this way, functional connectivity during the EO and EC condition was determined, along with the difference in connectivity between these two states (EO>EC). As age has been observed to affect functional connectivity measures [55], [56], subject age was included in all group-level analyses.

Target ROIs were converted from standard space into each subject’s functional space using previously calculated linear transformations. Mean z-values for the functional connectivity between the mPFC and target ROIs were then extracted.

### DTI

Diffusion-weighted images were acquired using a body coil transmit and a 32-channel receive headcoil. Sixty-four slices were aligned parallel to the AC-PC axis and data acquired for 99 diffusion weighting directions with a resolution of 1.9×1.9×1.9 mm^3^ (FOV = 243×243 mm^2^, matrix = 128×128, slice thickness = 1.9 mm, TE = 89 ms, TR = 8300 ms, Fourier factor = 6/8, 99 acquisitions with b = 1000 s/mm^2^, 10 acquisitions with b = 0). In addition, a field map was acquired to correct for field distortions (FOV = 256×256 mm^2^, matrix = 128×128, slice thickness = 2 mm, TE_1_ = 5.09 ms, TE_2_ = 7.55 ms, TR = 1000 ms, Fourier factor = 6/8).

Raw data were corrected for field distortions, eddy current distortions, and motion artifacts using FSL’s FUGUE and eddy_correct tools. DTIFIT was then used to fit a diffusion tensor model at each voxel. Fiber tracking was carried out using Diffusion Toolkit [57] and an interpolated streamline approach with an angle threshold of 35°. Standard space ROIs were converted to DTI space using linear transformations and the tracks between the mPFC MRS ROI and each of the target ROIs visualised using Trackvis [57]. The number of tracks, and mean fractional anisotropy (FA) within tracks (weighted by track length), for each ROI pair was obtained from these images.

### Combination of Measures

For each target ROI, the mPFC to ROI FC z-values for each of the EO, EC, and EO>EC conditions were firstly correlated with the level of Glx/NAA in the mPFC, followed by Glu/NAA from the same region. All regions that showed significant correlations with mPFC Glu/NAA were then also tested with left insula Glu/NAA and mPFC Gln/NAA values to show both regional specificity (i.e., to show that the Glu/NAA correlation was specifically with mPFC Glu/NAA, and not with the same metabolite from other regions also) and biochemical specificity (i.e., to show that the correlation with mPFC glutamate was specific to that metabolite and was not also present with the chemically similar glutamine, which does not act as a neurotransmitter [58]). This approach of only testing those correlations with mPFC Glu/NAA that were significant for regional and biochemical specificity was taken in order to reduce the number of overall comparisons made. Where subjects had tracks linking the mPFC and a particular target ROI, FC z-values were, in a second step, correlated with both the number of tracks and the mean FA within tracks.

Partial non-parametric correlations (Spearman) were used throughout [59], taking into account subject age, mPFC ROI GM volume, and the proportion of the mPFC ROI in the relevant hemisphere. Individual GM volumes were calculated by applying subject mPFC ROIs to the segmented GM maps created using FAST, as described above. Significance was set at p<0.05 for all analyses and was calculated through permutation tests (10,000 repetitions). All statistical analyses were carried out in Octave (http://www.gnu.org/software/octave/).

## Results

Correlation details for functional connectivity with mPFC Glu/NAA and DTI measures are given in Table 1. Distributions of FC z-values and all scatter plots for mPFC Glu/NAA, and track numbers can be found in the supplementary material (Figures S3 & S4, respectively). No regions that showed correlations with mPFC Glx/NAA or Glu/NAA also showed correlations with either of the control mPFC Gln/NAA and left insula Glu/NAA measures (Table S2 for control correlation details).

**Table 1:** Overview of correlations.

	Glu/NAA	No. Tracts	Mean FA	Glu/NAA	No. Tracts	Mean FA
	L NAc			R NAc		
EO	−0.05 (0.83)	0.28 (0.34)	−0.44 (0.11)	0.66 (0.012)*	0.2 (0.5)	0.07 (0.81)
EC	0.28 (0.34)	−0.01 (0.97)	−0.42 (0.13)	0.48 (0.098)†	0.37 (0.21)	0.08 (0.79)
EO>EC	−0.40 (0.17)	0.25 (0.38)	−0.09 (0.77)	0.41 (0.16)	−0.32 (0.29)	−0.02 (0.94)
	**L DMT**			**R DMT**		
EO	0.40 (0.15)	−0.62 (0.08)†	0.38 (0.31)	0.59 (0.031)*	−0.94 (<0.001)*	0.97 (<0.001)*
EC	0.19 (0.53)	−0.61 (0.08)†	0.97 (<0.001)*	0.07 (0.82)	−0.84 (<0.001)*	0.97 (<0.001)*
EO>EC	0.52 (0.07)†	0.08 (0.83)	−0.45 (0.23)	0.67 (0.011)*	−0.68 (0.06)†	0.55 (0.15)
	**L PAG**			**R PAG**		
EO	−0.28 (0.34)	0.23 (0.5)	−0.4 (0.23)	−0.1 (0.74)	0.5 (0.14)	0.19 (0.59)
EC	−0.63 (0.021)*	0.05 (0.89)	−0.23 (0.49)	−0.72 0.0058)*	0.83 (0.003)*	−0.08 (0.83)
EO>EC	0.20 (0.50)	0.37 (0.27)	−0.48 (0.13)	0.67 (0.011)*	−0.47 (0.17)	0.29 (0.43)

Correlations between mPFC to target FC and mPFC Glu/NAA, number of connecting tracts, and mean tract FA, in the left and right NAc, DMT, and PAG. R−values are given with p-values in parenthesis.

* indicates statistical significance,

† denotes a trend to significance.

### NAc

FC values between the mPFC and the right NAc showed a trend towards a positive correlation with mPFC Glx/NAA (r = 0.47, p = 0.10) during the EO condition that became significant with Glu/NAA (r = 0.67, p = 0.012). There were no correlations with the EC condition (although a trend towards a significant positive correlation with Glu/NAA was observed – r = 0.48, p = 0.098), nor with the EO>EC difference. No correlations with mPFC Glx/NAA or Glu/NAA were seen in the left NAc during EO, EC, or EO>EC.

Tracks linking the mPFC and the right NAc were observed in twelve subjects and in thirteen with the left NAc, with a mean track number of 33.1 (±7.9 S.E.M) in the right NAc and 74.4 (±9.3 S.E.M.) in the left. No correlation was seen between FC values and any structural measures in either the right or left NAc, although a trend to significance was seen between EO FC and mean FA in the left NAc (r = −0.52, p = 0.068).

**Figure 2:**
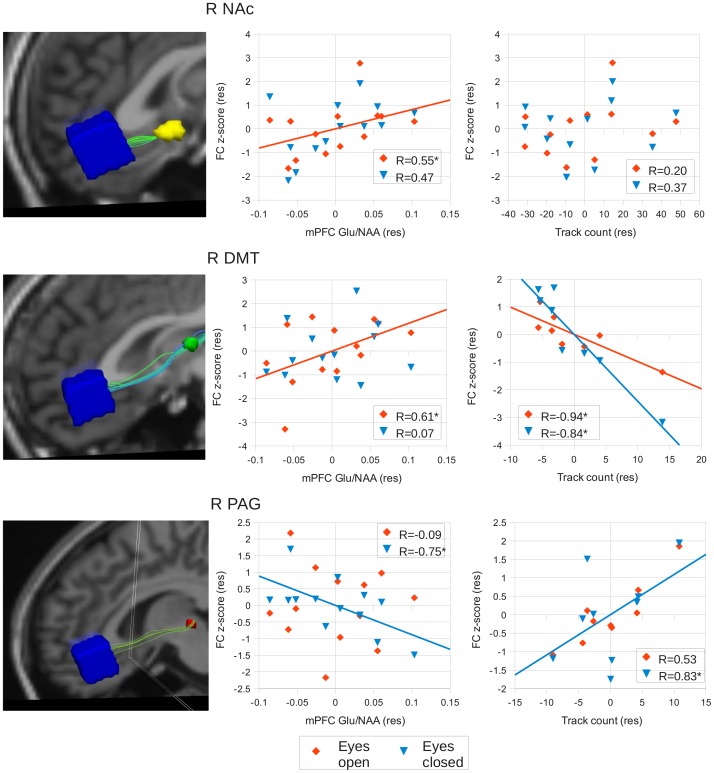
Correlation results between FC, Glu and DTI. Example tracts between the mPFC and each of the target regions are shown along with partial correlation graphs from the right hemisphere. Correlations between FC and Glu are shown, followed by correlations between FC and number of tracts. Note that values represent residuals after confounding variables have been regressed out of the data in the partial correlation (see Methods). Red diamonds = eyes open, blue triangles = eyes closed. * indicates p<0.05.

The degree of FC between the mPFC and right NAc during the EO condition was thus found to increase with increasing concentrations of mPFC glutamate. The relationship between FC and Glu/NAA was not found to be mirrored by a correlation between FC and the measures used of structural connectivity between the mPFC and NAc (see Figure 2).

### DMT

FC values between the mPFC and right DMT showed a trend towards a correlation with mPFC Glx/NAA (r = 0.48, p = 0.093), and were positively correlated with mPFC Glu/NAA (r = 0.59, p = 0.031), during the EO condition but not the EC condition. The EO>EC difference was also found to be positively correlated with mPFC Glu/NAA (r = 0.68, p = 0.011), with a trend to significance with Glx/NAA (r = 0.49, 0.089). No correlations were seen in the left DMT, although trends towards significance were seen with Glu/NAA and EO (r = 0.41, p = 0.16) and EO>EC (r = 0.52, p = 0.07) functional connectivity.

Eight out of thirteen subjects had tracks linking the mPFC and right DMT (mean track number = 22.4±6.09 S.E.M), whilst nine out of thirteen subjects had tracks linking the mPFC and left DMT (mean track number = 57.9±13.7 S.E.M). FC values in the right DMT were found to negatively correlate with the number of tracks present, as well as positively with the mean FA within these tracks, during the EO and EC conditions. No relationship between EO>EC FC difference and any structural measure was observed in this region. No correlation was seen between FC in any condition and any of the biochemical or structural measures in the left DMT (see Table 1).

As with the NAc, the degree of FC between the mPFC and right DMT during the EO condition was seen to increase with increasing concentrations of mPFC Glu/NAA. In addition, the difference in FC between the EO and EC conditions was also found to increase with increasing Glu concentrations. This relationship was mirrored by a correlation between EO FC and structural measures, with the degree of FC decreasing with a greater number of tracks and increasing as track FA increases (see Figure 2).

### PAG

Right PAG FC correlated negatively with mPFC Glx/NAA (r = −0.62 p = 0.024) and Glu/NAA in the EC condition (r = −0.75, p = 0.0025). Positive correlations between the EO>EC difference and Glx/NAA (r = 0.61, p = 0.026) and Glu/NAA were also seen (r = 0.7, p = 0.0053). EC FC in the left PAG was found to correlate negatively with mPFC Glu/NAA (r = −0.64, p = 0.014).

Nine out of thirteen subjects had tracks linking the mPFC and right PAG (mean track number = 6.2±1.98 S.E.M), with the same number having tracks linking the mPFC and left PAG (mean track number = 14.36±6.21 S.E.M). Right PAG EC FC was positively correlated with track number (r = 0.83, p = 0.0028), although no relationship was seen with FA values. No correlations between left PAG FC and structural measures were observed (Table 1).

In summary, the degree of FC between the mPFC and right PAG during the EC condition decreased with greater mPFC Glu/NAA concentrations. With an increase in the number of tracks between the mPFC and PAG there was an increase in the degree of FC (see Figure 2). The same indirect relationship between Glu/NAA and track number in relation to FC was thus seen in the PAG as in the DMT (i.e., lower track counts with higher Glu/NAA).

## Discussion

**Figure 3:**
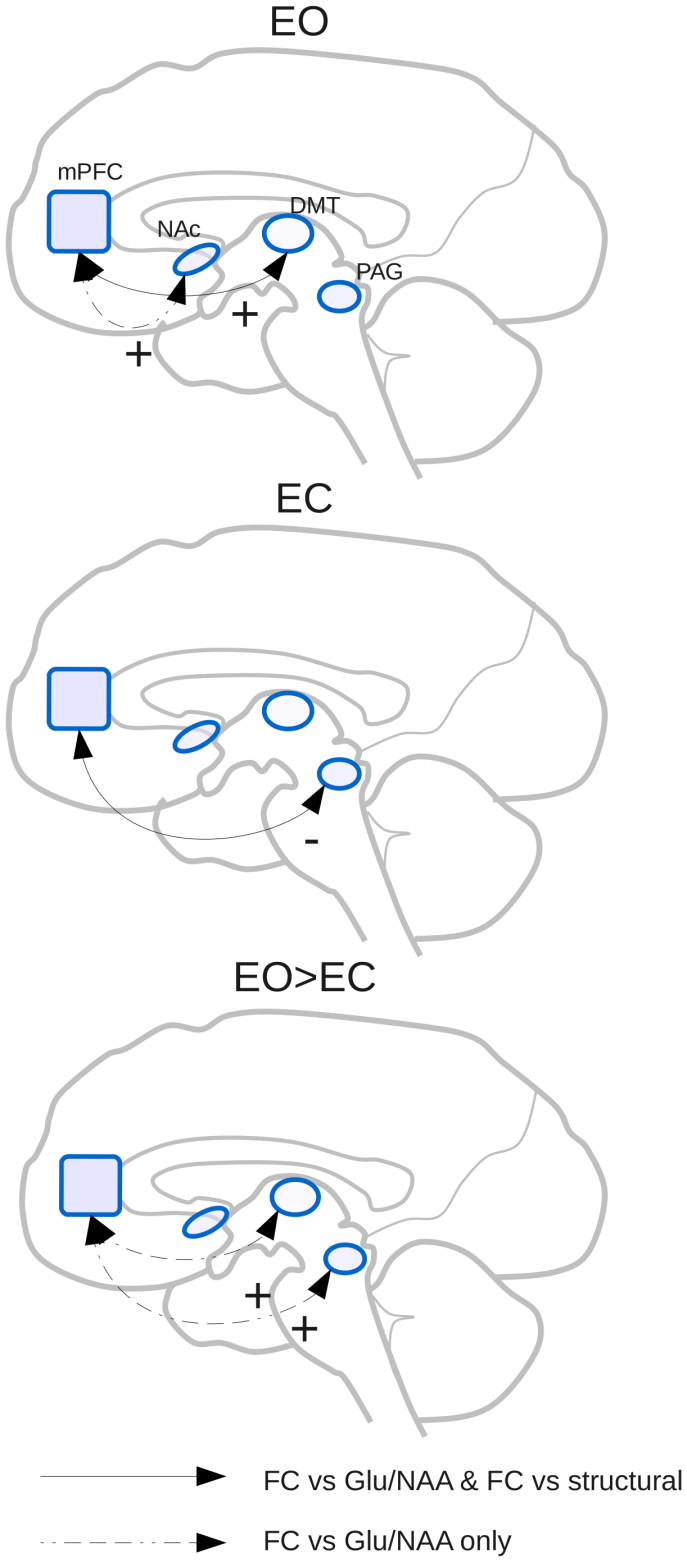
Result overview. Overview of connectivity in EO, EC and EO>EC conditions. Shown are the mPFC, NAc, DMT, and PAG. Black lines denote that a relationship between FC and both Glu/NAA and structural measures are seen in the relevant condition. Dashed lines denote that a relationship between FC and Glu/NAA only is seen.+symbol beside arrow denotes a positive correlation with Glu/NAA, - symbol beside arrow denotes a negative correlation with Glu/NAA.

We here show that the concentration of glutamate in the mPFC predicted the degree of FC between this region and various subcortical regions (the right NAc, DMT, and PAG). FC was also found to correlate with measures of structural connectivity between the mPFC and the DMT and PAG, but not NAc, hence providing evidence that the FC with these regions may be a result of direct anatomical communication (see Figure 3 for an overview of results). The inclusion of the left insula as a control region further supports the regional specificity of the results. These findings show, for the first time in humans, that the strength of cortical-subcortical connectivity is associated, in part, with cortical glutamate concentrations. This complements previous studies using non-human animals (see below) and may have important implications for our understanding of brain networks and neuropsychiatric disorders.

### mPFC Glutamate and Subcortical Functional Connectivity

Both structural [25], [32], [60], [61] and functional connectivity [54], [62], [63] between the mPFC and each of the subcortical target regions has been demonstrated previously through a combination of human and non-human imaging and postmortem studies. The mPFC itself is rich in glutamatergic cells and innervation [64], [65] and has been identified as an important site for the mediation of the behavioural effects of glutamatergic agents [66]. In both humans and animals, mPFC task-related activity is altered when glutamatergic agents (such as ketamine) are administered [26], [67]–[71], as is functional connectivity between the mPFC and other brain regions [24], [69]. The current findings thus fit well into the patterns of connectivity and biochemistry known for the mPFC. In addition, these previous findings lend weight to the conclusion that the relationship between Glu/NAA and FC seen here is at least partly related to direct connectivity in the PAG and DMT (the regions that show a correlation between FC and structural measures).

Glutamate has also been shown to play in important role in the function of each of the target regions (often in conjunction with other transmitters). In the NAc, glutamatergic inputs come from multiple cortical and subcortical sites, including the mPFC [25], [37], [72]. The role of glutamate within the NAc in relation to behaviour has been well established, as has the effect of glutamatergic agents on its biochemical functioning, such as on dopamine release, and on it’s effect on behaviour, such as on movement arousal [6], [18], [66]. Important in this modulation of NAc functioning appears to be the interaction between the mPFC and NAc [73], where glutamatergic communication plays a key role [6], [66], [74]. Glutamate also plays a role in communication between the DMT and mPFC, as is demonstrated in a number of animal studies, and by glutamatergic substances producing correlated changes in the activity of each region [24], [26], [75], [76]. Within the DMT itself, glutamate is a key neurotransmitter [75]. Finally, the mPFC has been identified as an important source of glutamateric projections to the PAG [22], [33], [77]. These connections appear to be important for the cortical modulation of pain and fear responses mediated by the PAG [22], [63].

Resting-state functional connectivity itself is a measure of the temporal synchronisation between the activity in different brain regions. In humans, FC studies have focused on a range of frequencies, from spontaneous BOLD fluctuations in low frequency ranges [78], [79] to higher frequency ranges in EEG [80], [81]. The role of glutamate in functional connectivity in humans has not been widely studied to date, although some preliminary studies have been carried out [10], [82]. In animals, both ionotropic NMDA and metabotropic glutamate receptors have been shown to be involved in brain-wide synchronisation of activity at a range of fluctuation frequencies [83]–[85], although it is worth noting that this action is likely to be in conjunction with other transmitters, such as GABA [86]. In addition, glutamatergic agents like phencyclidine and ketamine have been shown to reduce cortical synchrony in humans [69], [75].The current study thus fits into a background of evidence that glutamate is involved in the temporal synchronisation between regions across the brain and builds on it by demonstrating such a role in relation to specific regions.

The correlations present between mPFC Glu and FC during the EC condition no longer held during EO, and *vice versa*. Such changes in brain activity properties between the EO and EC resting-state have been described previously in humans [87]–[92]. Interestingly, not all of the changes observed in such studies are due to a simple increase in visual stimulation in EO, as changes are also seen in complete darkness and in congenitaly blind subjects [91], [93], [94]. Compared to the EC condition, EO induces a reduction in global alpha activity, along with an increase in skin-conductance [95], [96], increased activity in the DMT [88] and a brain-wide (although mPFC focused) increase in FC [81]. Such changes, particularly those in alpha activity, are likely to be linked to an increase in arousal or attention (broadly construed) as the eyes are opened, with a concurrent change in functional organisation across the brain [81], [95], [97]. Given this, one could hypothesise that the changes in the relationship between mPFC Glu and FC between EO and EC seen here are due in part to a switching in arousal or attention, and as such in the organisation of neural activity, as the eyes are opened or closed. This would be in line with the established role for the mPFC in the modulation of arousal in humans [98], [99] and with mPFC glutamate-related modulation of arousal in rats [66], [100]. Such a process would also provide a possible explanation for the notable right-lateralisation of the current NAc and thalamus results, as previous studies have shown the importance a strongly right-lateralised network in arousal and attention, that includes the mPFC, thalamus and striatum, in arousal and attention [88], [101]. Research with direct measures of arousal, such as skin conductance, are however required to explore this possibility further.

Also notable in the context of EO/EC differences is that the NAc and DMT correlate positively with mPFC Glu/NAA during EO, but that the PAG correlates negatively during EC. This difference may be a result of mPFC Glu/NAA levels being associated with regulating arousal-related activity in a positive direction in the EO condition but being more involved in a down-regulation of PAG activity in the EC condition, suppressing this regions more externally-oriented functions in line with the EC reduction in arousal [35], [102]. This suggestion is supported by positive functional connectivity between the mPFC and PAG in an EO condition having been shown in humans [48], providing an elevated starting position from which down-regulation may occur in EC. Were this to be the case, higher Glu/NAA levels in the mPFC would presumably be associated with a greater reduction in FC between EO and EC. This is indeed what is seen here, with a positive correlation between mPFC Glu/NAA and PAG EO>EC FC differences. The contrast between the hypothesised positive role for glutamate in the case of EO DMT/NAc FC and a more inhibitory role in the case of EC PAG FC highlights the complex roles that this transmitter plays in the brain. Further research that more closely targets different glutamate receptors (e.g., NMDA vs metabotropic) is required to explore these roles, potentially through novel PET ligands or through pharmacological challenge.

### Implications for MDD and Schizophrenia

Both major depressive disorder (MDD) and schizophrenia have been related to changes in glutamatergic function [16], [103], [104] and to altered activity in the mPFC [15], [105]. In the case of MDD, hyperactivity in the mPFC during the resting state has been described in humans and animals, with this hyperactivity being concurrent with hyperactivity in a set of subcortical regions that includes the NAc, DMT, and PAG [14], [105]. In addition to these activity changes in MDD, changes in mPFC Glu and glutamate receptors in MDD have been observed in humans and animals [106]–[109]. In this context, our findings that mPFC glutamate is related to FC between the mPFC and subcortical regions is suggestive of a role for this transmitter in altered cortical-subcortical regulation in MDD. The anti-depressant effects of glutamatergic substances such as ketamine could then be hypothesised to involve a rebalancing action on such alterations [16].

As with MDD, glutamatergic alterations and the action of glutamatergic substances have been linked to schizophrenia [17]. In the mPFC specifically, changes in glutamatergic receptors and uptake proteins have been reported [103], [104]. However, conversely to MDD, mPFC resting activity appears to be decreased in schizophrenia [15], [110].These mPFC changes have then been suggested to impact upon cortico-striatal-thalamic loops, producing imbalances in brain networks that lead to the symptoms of the condition [111], [112]. The involvement of the thalamus implied by such modulation of cortico-striatal-thalamic loops links with the current findings and is supported by a wide range of evidence [36], including structural and biochemical [36], [112], [113], as well as changes in resting-state FC [114]. In addition to the thalamus, striatum involvement in schizophrenia, and specifically the NAc, has been indicated by altered cortico-striatal dopamine regulation, with this in turn linked to schizophrenic-type symptoms [115], [116]. The connection made here between glutamate and cortico-striatal communication could thus be taken in the context of mPFC-NAc glutamate-dopamine interaction [6], [66], [74] to provide a tentative link between the roles of these transmitters in schizophrenia. As with MDD, then, the current findings fit into a pathology scenario that involves dysregulation of cortical-subcortical networks and dysfunctional glutamatergic communication [14], [117] and as such point towards potentially interesting avenues of research into glutamate-related cortico-subcortical network function in humans.

### Limitations & Conclusion

A number of limitations of the current study must be taken into account. Firstly it is worth noting that the mPFC region studied was quite large (to allow the use of the MEGA-PRESS method), being mainly centred upon the pgACC, extending forward to include a portion of BA 10, and also covering the white matter lateral to the ACC. The anatomical specificity of the results is therefore somewhat limited. Though the current interpretation may benefit from future studies using smaller MRS regions (when feasible) or higher fields (like 7 T), the present findings are nonetheless largely supported by findings in both the human and animal literature. Similarly, with the current resolution we cannot differentiate between, for example, the shell and the core of the NAc, or different DMT nuclei. The lack of resolution in the NAc may explain the dissociation between the glutamate and DTI results through the differences in innervation between these subdivisions [25], [118], although this is speculative. Secondly, FC is not a directional or causative measure, nor does it give any information about the relative intensity of activity in different regions. Instead it gives information only about whether the BOLD signal is more or less synchronised with that in the seed region. These features mean that one must be hesitant in making too strong a claim about the meaning of any FC result. For example, although in the current study a relationship between mPFC Glu/NAA and NAc FC is seen, the NAc also receives input from the DMT [119]. The relationship seen in the NAc may therefore be due to an indirect synchronisation in the BOLD signal mediated by a third region, such as the DMT (or indeed by a region with input to both seed and target not analysed here, such as the ventral tegmental area [72], [120]). Finally, the final study group was somewhat small as the data from a number of participants had to be rejected due to problems with either head-movement or with spectral quality.

To conclude, a role for mPFC glutamate in functional connectivity between the cortex and subcortical regions (specifically the NAc, DMT, and PAG) in humans was shown. In the DMT and PAG this was mirrored by a correlation between FC and structural connectivity measures, suggesting that the glutamatergic relationship is due to direct anatomical connectivity in at least these regions. These results extend existing results from non-human animal studies and may have relevance to a range of psychiatric conditions, including MDD and schizophrenia.

## Supporting Information

Figure S1 **MRS voxel location and spectra.** (a) mPFC (blue) and left insula (red) MRS voxel locations shown on the MNI template. (b) LCModel quantification of the representative 1H NMR spectrum obtained using MEGA-PRESS sequence from a 48×21×21 mm^3^ vovel placed in the mPFC region of the human brain. The contribution of Glu and Gln are shown.
(PDF)

Figure S2 **Resting-state scan design.** (a) Subjects carried out alternating 120 s blocks of EO and EC resting-state, as illustrated. (b) Sample fMRI analysis design showing mPFC timecourse for EO and EC, along with eight nuisance regressors (6×head motion (HM), white matter (WM) timecourse, and cerebro-spinal fluid (CSF) timecourse).
(PDF)

Figure S3 **Resting-state FC distribution.** Distribution of mPFC to target FC z-scores for each region. Each point represents one subject. Unfilled circles = left hemisphere, filled circles = right hemisphere.
(PDF)

Figure S4 **FC vs mPFC Glu/NAA & tract count.** (a) Scatter plots for FC for all regions vs mPFC Glu/NAA in EO, EC and EO>EC conditions. Note that values are residuals following the removal of confounding variables. (b) Scatter plots for FC for all regions vs tract count in EO, EC and EO>EC conditions. Note that values are residuals following the removal of confounding variables.
(PDF)

Table S1 **Metabolite values.** MRS values for mPFC and left insula. Metabolite concentrations in relation to NAA are given. Cramér-Rao lower bounds (%SD) for Glx, Glu, and Gln are shown in square brackets.
(PDF)

Table S2 **Overview of control correlations.** Regional and biochemical specificity controls. Correlations between mPFC to target FC and mPFC Gln/NAA+left insula Glu/NAA.
(PDF)
