# Correction: Glutamate Concentration in the Medial Prefrontal Cortex Predicts Resting-State Cortical-Subcortical Functional Connectivity in Humans

**DOI:** 10.1371/annotation/8a1feb12-d70a-4fb2-8dcb-a9cad56c3afd

**Published:** 2013-06-21

**Authors:** Niall W. Duncan, Christine Wiebking, Brice Tiret, Malgoranza Marjańska, Dave J. Hayes, Oliver Lyttleton, Julien Doyon, Georg Northoff

The name of the fourth author is incorrect. The correct name is: Malgorzata Marjańska.

