# Supplementary material for: Glutamate Concentration in the Medial Prefrontal Cortex Predicts Resting-State Cortical-Subcortical Functional Connectivity in Humans
Source: PLoS One. 2013 Apr 3;8(4):e60312. doi: 10.1371/journal.pone.0060312 (PMC3616113; doi:10.1371/journal.pone.0060312)
Supplement: Figure S1 — MRS voxel location and spectra. (a) mPFC (blue) and left insula (red) MRS voxel locations shown on the MNI template. (b) LCModel quantification of the representative 1H NMR spectrum obtained using MEGA-PRESS sequence from a 48×21×21 mm3 vovel placed in the mPFC region of the human brain. The contribution of Glu and Gln are shown. (PDF) [file pone.0060312.s001.pdf]

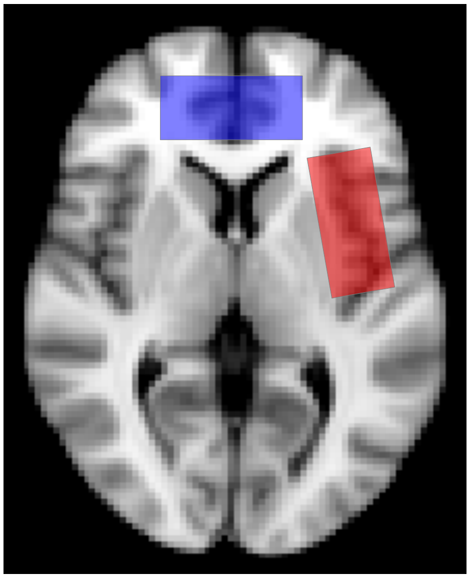

Supplemetary figure 1A: mPFC (blue) and left insula (red) MRS voxel locations shown on the MNI template.

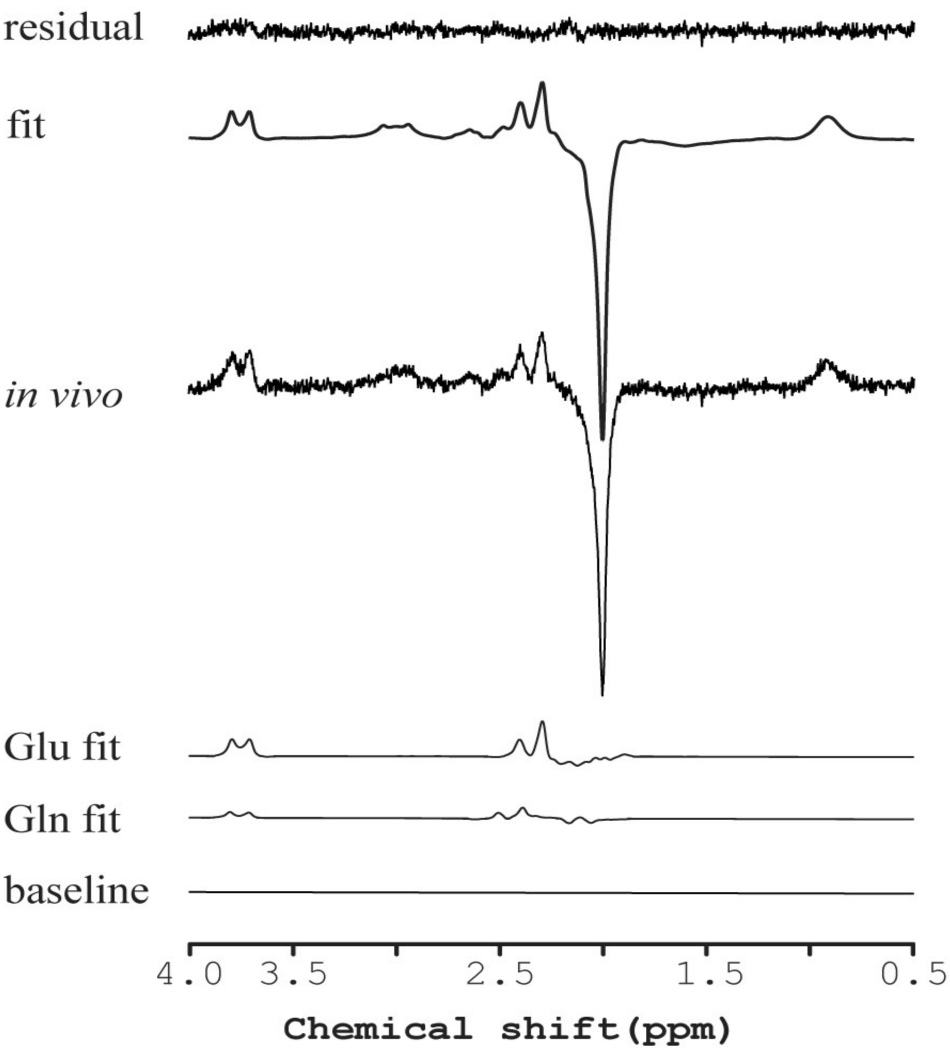

Supplemetary figure 1B: LCModel quantification of the representative 1H NMR spectrum obtained using MEGA-PRESS sequence from a 48 x 21 x 21 mm^3 vovel placed in the mPFC region of the human brain. The contribution of Glu and Gln are shown.
