# Supplementary material for: Glutamate Concentration in the Medial Prefrontal Cortex Predicts Resting-State Cortical-Subcortical Functional Connectivity in Humans
Source: PLoS One. 2013 Apr 3;8(4):e60312. doi: 10.1371/journal.pone.0060312 (PMC3616113; doi:10.1371/journal.pone.0060312)
Supplement: Figure S2 — Resting-state scan design. (a) Subjects carried out alternating 120 s blocks of EO and EC resting-state, as illustrated. (b) Sample fMRI analysis design showing mPFC timecourse for EO and EC, along with eight nuisance regressors (6×head motion (HM), white matter (WM) timecourse, and cerebro-spinal fluid (CSF) timecourse). (PDF) [file pone.0060312.s002.pdf]

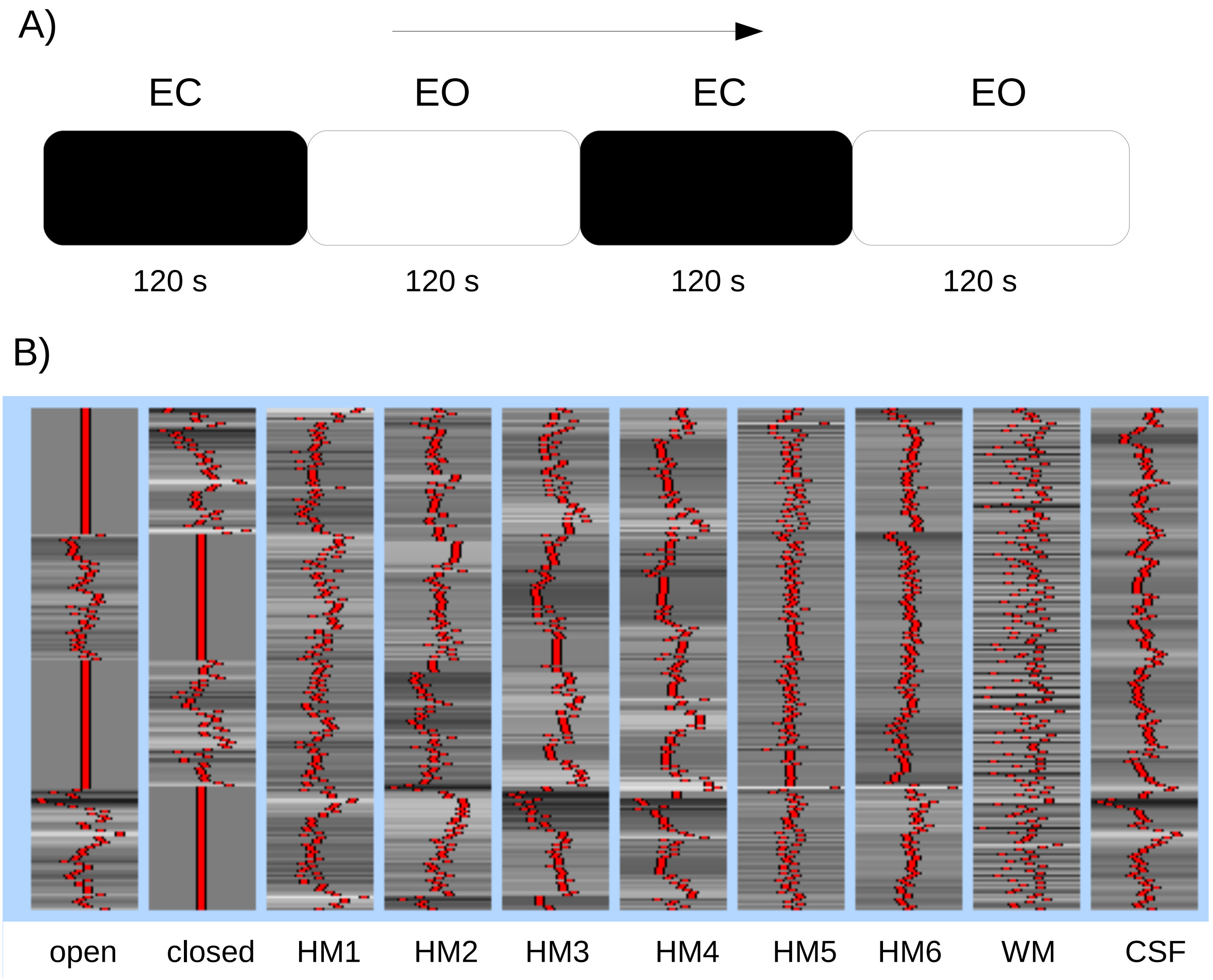

Supplementary figure 2: A) Subjects carried out alternating 120 s blocks of EO and EC resting-state. B) Sample fMRI analysis design showing mPFC timecourse for EO and EC, along with eight nuisance regressors (6 x head motion (HM), white matter (WM) timecourse, and cerebro-spinal fluid (CSF) timecourse).
