# Supplementary material for: Glutamate Concentration in the Medial Prefrontal Cortex Predicts Resting-State Cortical-Subcortical Functional Connectivity in Humans
Source: PLoS One. 2013 Apr 3;8(4):e60312. doi: 10.1371/journal.pone.0060312 (PMC3616113; doi:10.1371/journal.pone.0060312)
Supplement: Figure S3 — Resting-state FC distribution. Distribution of mPFC to target FC z-scores for each region. Each point represents one subject. Unfilled circles = left hemisphere, filled circles = right hemisphere. (PDF) [file pone.0060312.s003.pdf]

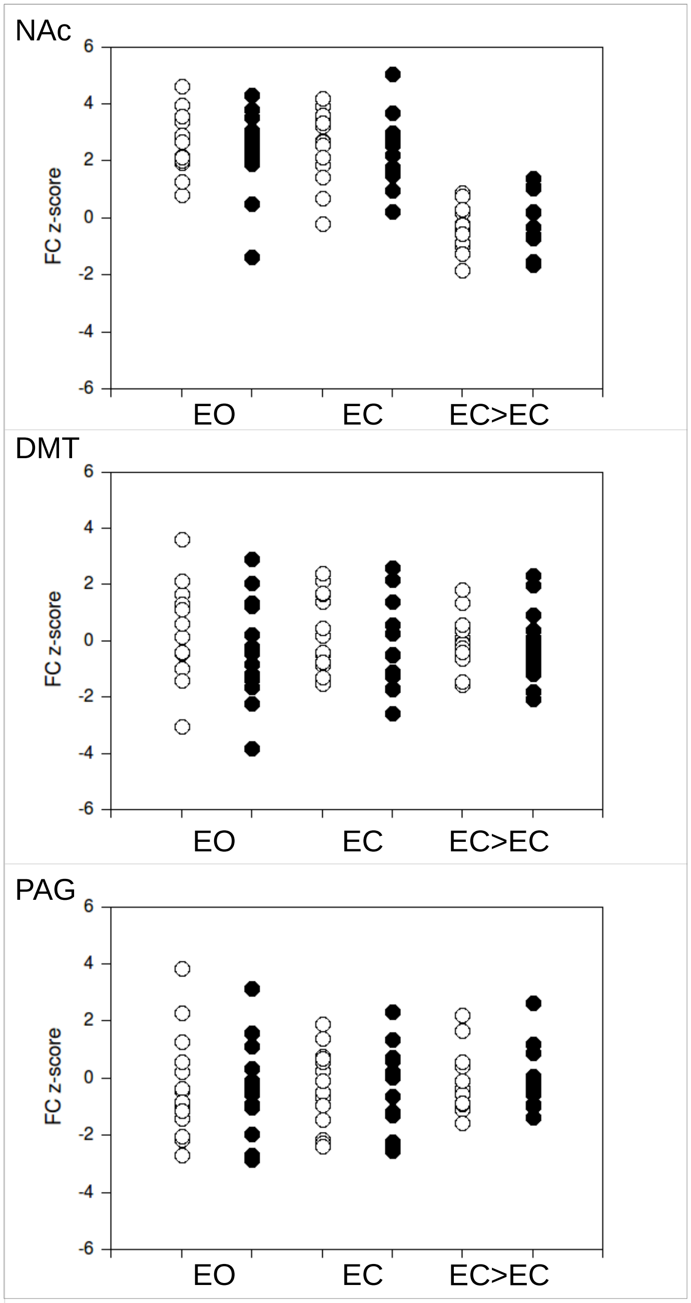

Supplementary figure 3: Distribution of mPFC to target FC z-scores for each region. Each point represents one subject. Unfilled circles = left hemisphere, filled circles = right hemisphere.
