# Supplementary material for: Glutamate Concentration in the Medial Prefrontal Cortex Predicts Resting-State Cortical-Subcortical Functional Connectivity in Humans
Source: PLoS One. 2013 Apr 3;8(4):e60312. doi: 10.1371/journal.pone.0060312 (PMC3616113; doi:10.1371/journal.pone.0060312)
Supplement: Figure S4 — FC vs mPFC Glu/NAA & tract count. (a) Scatter plots for FC for all regions vs mPFC Glu/NAA in EO, EC and EO>EC conditions. Note that values are residuals following the removal of confounding variables. (b) Scatter plots for FC for all regions vs tract count in EO, EC and EO>EC conditions. Note that values are residuals following the removal of confounding variables. (PDF) [file pone.0060312.s004.pdf]

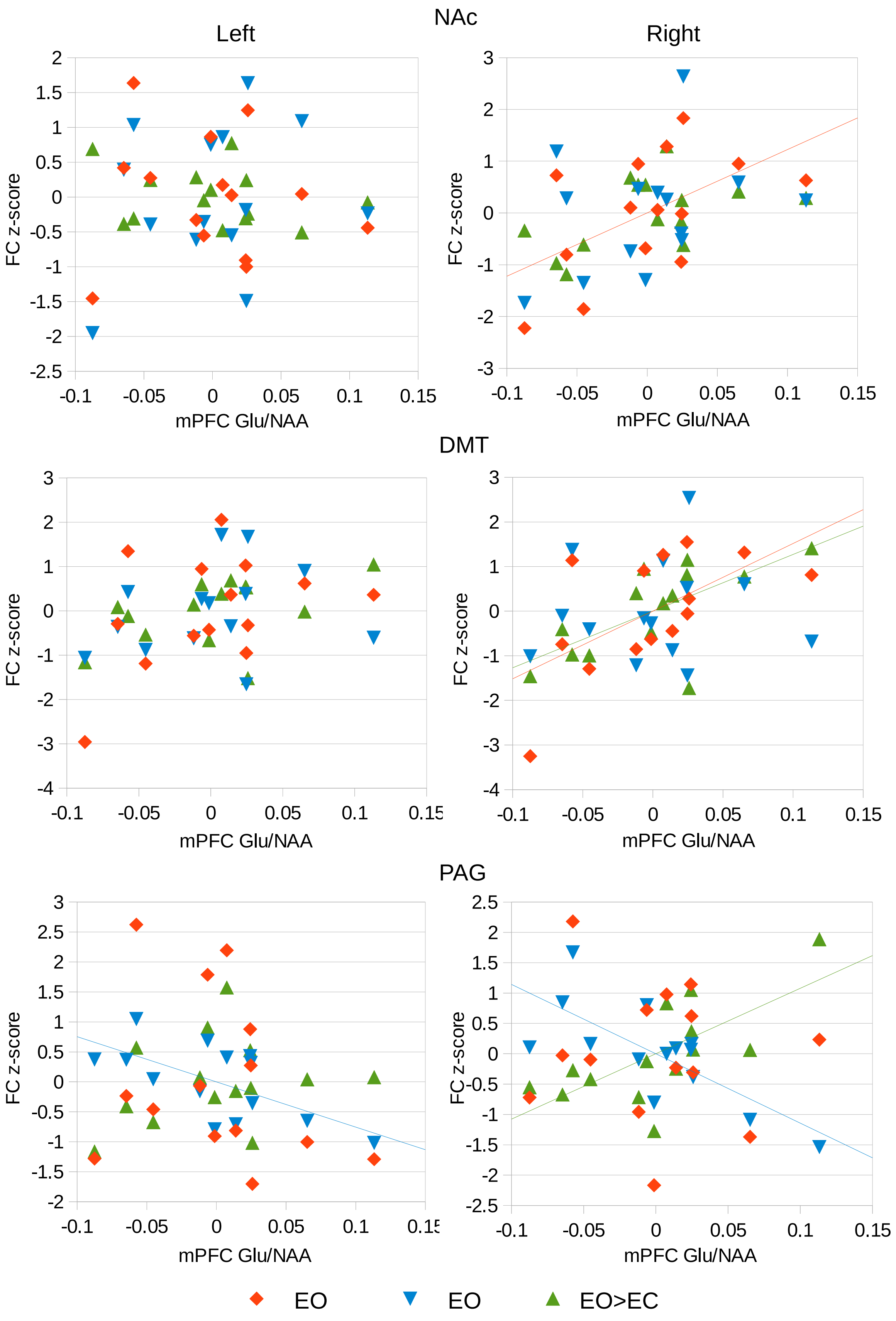

Supplementary figure 4a: Scatter plots for FC for all regions vs mPFC Glu/NAA in EO, EC and EO>EC conditions. Note that values are residuals following the removal of confounding variables.

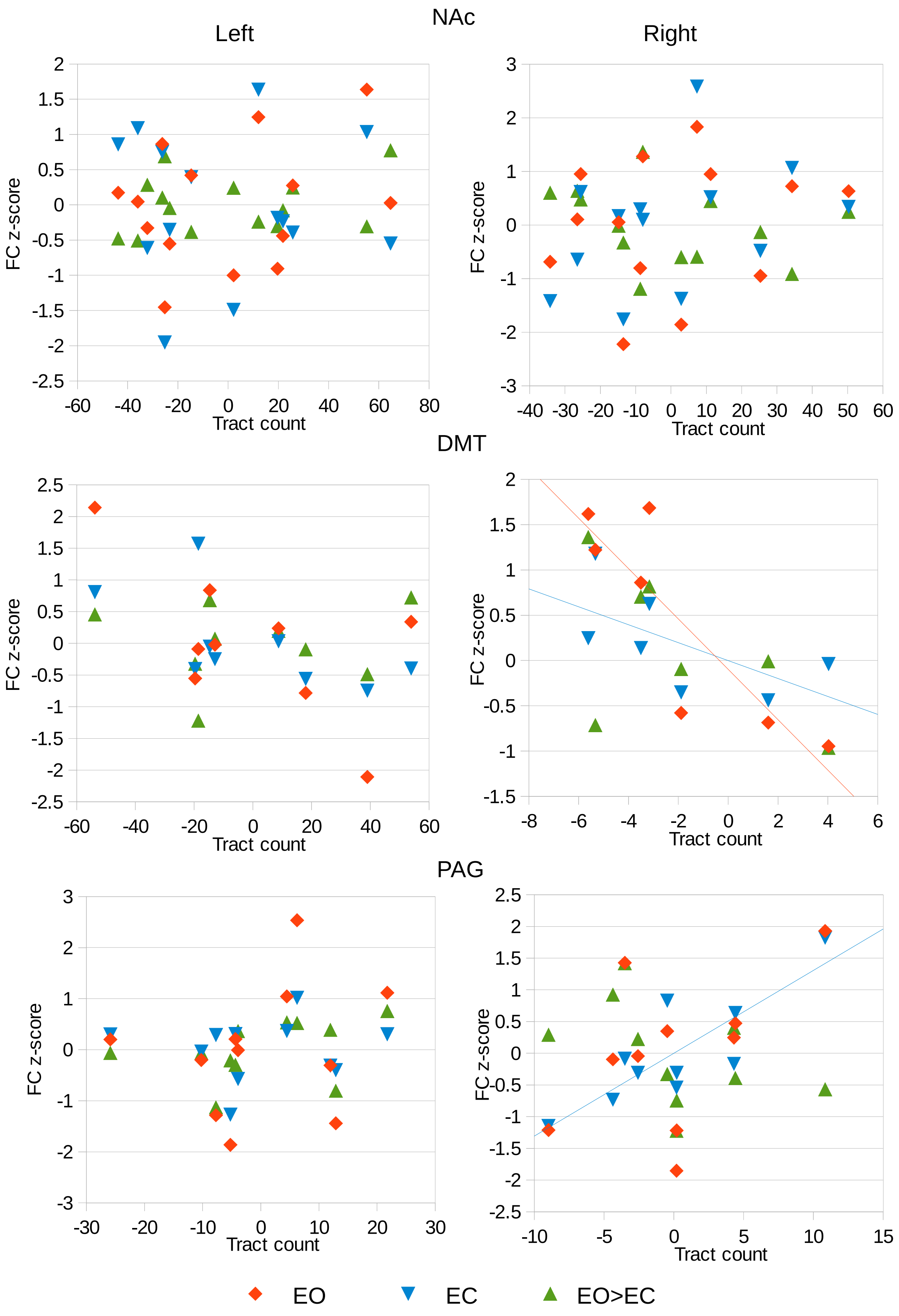

Supplementary figure 4b: Scatter plots for FC for all regions vs tract count in EO, EC and EO>EC conditions. Note that values are residuals following the removal of confounding variables.
