# Supplementary material for: Glutamate Concentration in the Medial Prefrontal Cortex Predicts Resting-State Cortical-Subcortical Functional Connectivity in Humans
Source: PLoS One. 2013 Apr 3;8(4):e60312. doi: 10.1371/journal.pone.0060312 (PMC3616113; doi:10.1371/journal.pone.0060312)
Supplement: Table S1 — Metabolite values. MRS values for mPFC and left insula. Metabolite concentrations in relation to NAA are given. Cramér-Rao lower bounds (%SD) for Glx, Glu, and Gln are shown in square brackets. (PDF) [file pone.0060312.s005.pdf]

	mPFC			Left insula		
subject	Glx/NAA	Glu/NAA	Gln/NAA	Glx/NAA	Glu/NAA	Gln/NAA
1	0.884 [3]	0.625 [5]	0.259 [16]	0.911 [3]	0.692 [5]	0.219 [18]
2	0.953 [3]	0.736 [4]	0.216 [18]	0.952 [1]	0.715 [2]	0.237 [8]
3	0.974 [2]	0.734 [3]	0.240 [10]	0.920 [2]	0.719 [3]	0.201 [11]
4	0.927 [3]	0.709 [4]	0.218 [13]	0.914 [2]	0.734 [4]	0.180 [17]
5	0.949 [2]	0.729 [3]	0.220 [12]	0.860 [2]	0.654 [3]	0.206 [9]
6	1.056 [2]	0.762 [4]	0.293 [11]	0.727 [3]	0.597 [3]	0.130 [17]
7	0.915 [2]	0.738 [3]	0.177 [16]	0.750 [2]	0.569 [3]	0.181 [11]
8	1.065 [2]	0.740 [3]	0.325 [7]	0.952 [3]	0.675 [4]	0.277 [12]
9	0.925 [3]	0.617 [9]	0.308 [9]	0.815 [3]	0.627 [3]	0.188 [12]
10	1.045 [3]	0.815 [5]	0.230 [20]	0.829 [2]	0.656 [3]	0.173 [12]
11	0.913 [2]	0.627 [4]	0.286 [12]	0.835 [3]	0.650 [4]	0.185 [16]
12	1.086 [2]	0.782 [3]	0.304 [10]	0.921 [2]	0.716 [3]	0.205 [11]
13	0.893 [2]	0.718 [3]	0.175 [13]	0.987 [2]	0.719 [4]	0.268 [13]
Mean (SD)	0.968 (0.07)	0.718 (0.06)	0.250 (0.05)	0.875 (0.08)	0.671 (0.05)	0.204 (0.04)

Supplementary table 1: MRS values for mPFC and left insula. Metabolite concentrations in relation to NAA are given. Cramér-Rao lower bounds (%SD) for Glx, Glu, and Gln are shown in square brackets.
