# Supplementary material for: Glutamate Concentration in the Medial Prefrontal Cortex Predicts Resting-State Cortical-Subcortical Functional Connectivity in Humans
Source: PLoS One. 2013 Apr 3;8(4):e60312. doi: 10.1371/journal.pone.0060312 (PMC3616113; doi:10.1371/journal.pone.0060312)
Supplement: Table S2 — Overview of control correlations. Regional and biochemical specificity controls. Correlations between mPFC to target FC and mPFC Gln/NAA+left insula Glu/NAA. (PDF) [file pone.0060312.s006.pdf]

	mPFC Gln/NAA	Insula Glu/NAA	mPFC Gln/NAA	Insula Glu/NAA
	**L NAc**		**R NAc**	
EO	0.25(0.40)	0.23 (0.41)	0.05 (0.86)	0.14 (0.61)
EC	0.37 (0.20)	0.4 (0.15)	0.05 (0.84)	0.4 (0.15)
EO>EC	-0.01 (0.97)	-0.63 (0.015)*	0.16 (0.61)	-0.55 (0.038)*
	**L DMT**		**R DMT**	
EO	-0.1 (0.72)	0.08 (0.78)	-0.09 (0.75)	0.08 (0.79)
EC	0.14 (0.64)	0.23 (0.42)	0.15 (0.61)	0.31 (0.27)
EO>EC	-0.29 (0.3)	-0.13 (0.65)	-0.19 (0.51)	-0.17 (0.56)
	**L PAG**		**R PAG**	
EO	0.21 (0.48)	0.07 (0.8)	0.05 (0.85)	-0.02 (0.94)
EC	0.17 (0.54)	0.18 (0.54)	0.01 (0.99)	0.19 (0.51)
EO>EC	0.15 (0.6)	0.01 (0.97)	0.01 (0.97)	-0.15 (0.6)

Supplementary table 2: Regional and biochemical specificity controls. Correlations between mPFC to target FC and mPFC Gln/NAA + left insula Glu/NAA.
